# Impact of rosuvastatin on the memory potential and functionality of CD8^+^ T cells from people with HIV

**DOI:** 10.1016/j.ebiom.2025.105672

**Published:** 2025-03-29

**Authors:** Federico Perdomo-Celis, Caroline Passaes, Valérie Monceaux, Olivier Lambotte, Dominique Costagliola, Mathieu F. Chevalier, Laurence Weiss, Asier Sáez-Cirión

**Affiliations:** a Institut Pasteur, Université Paris Cité, Viral Reservoirs and Immune Control Unit, Paris, 75015, France; b Institut Pasteur, Université Paris Cité, HIV Inflammation and Persistance Unit, Paris, 75015, France; c Instituto de Genética Humana, Facultad de Medicina, Pontificia Universidad Javeriana, Bogotá, Colombia; d Université Paris-Saclay, Inserm, CEA, Center for Immunology of Viral, Auto-immune, Hematological, Bacterial Diseases (IMVA-HB/IDMIT/UMRS1184), Le Kremlin Bicêtre, Fontenay aux Roses, France; e Assistance Publique Hôpitaux de Paris, Groupe Hospitalier Universitaire Paris Saclay, Service de Médecine interne immunologie clinique, Le Kremlin Bicêtre, France; f Sorbonne Université, INSERM, Institut Pierre Louis d'Épidémiologie et de Santé Publique (IPLESP), Paris, France; g INSERM UMR 1342, Institut de Recherche Saint-Louis (IRSL), Université Paris Cité, Paris, France; h Université de Paris Cité, AP-HP, Paris Centre, Paris, France

**Keywords:** HIV-1, CD8^+^ T cells, Statins, HIV control, HIV remission, T cell reprogramming

## Abstract

**Background:**

Virus-specific CD8^+^ T cells play a major role in the natural control of HIV infection, linked to memory-like features such as high survival capacity and polyfunctionality. However, virus-specific CD8^+^ T cells from HIV non-controllers exhibit an effector-like and exhausted profile, with limited antiviral potential. Metabolic reprogramming of cells from non-controllers could reinvigorate their functional capacities. Considering the implication of the cholesterol pathway in the induction of T cell exhaustion, here we evaluated the impact of rosuvastatin, an inhibitor of cholesterol synthesis, on the functionality and memory profile of HIV-specific CD8^+^ T cells from people on antiretroviral treatment.

**Methods:**

We analysed samples from 10 individuals with HIV-1 on ART who participated in the IMEA 043-CESAR trial and received rosuvastatin for 12 weeks. We explored whether rosuvastatin treatment was accompanied by changes in the memory potential of CD8^+^ T cells. We evaluated the phenotype and functionality of total and HIV-specific CD8^+^ T cells before, during, and after treatment with rosuvastatin. A mixed effects model was used for repeated measures and corrected for multiple comparisons.

**Findings:**

Total and HIV-specific CD8^+^ T cell survival and functionality were enhanced in individuals who received a 12-week course of rosuvastatin, with a consistent increase in polyfunctional IFN-γ^+^ TNF-α^+^ cells. The superior CD8^+^ T cell functionality after rosuvastatin treatment was associated with intrinsic metabolic changes, including the decrease of fatty acid uptake, as well as a reduction in effector/exhaustion markers. Changes in the characteristics of CD8^+^ T cells coincided with the duration of rosuvastatin administration, and most effects waned after the cessation of the treatment.

**Interpretation:**

CD8^+^ T cell metabolic reprogramming by targeting the cholesterol pathway, combined with other available immunotherapies, might represent a promising strategy in the search for the cure of HIV or other chronic viral infections.

**Funding:**

The CESAR trial was sponsored by 10.13039/501100010431IMEA. This work was supported by the NIH (grants UM1AI164562 and R01DK131476).

**Research in context**

**Evidence before this study**

A major objective in HIV remission strategies is to enhance CD8^+^ T cell responses so that they can efficiently eliminate HIV-infected cells and/or contribute to durably control viraemia without antiretroviral treatment (ART). Differences in the capacity of HIV-specific CD8^+^ T cells from HIV natural controllers and people who require ART to control infection are related, at least partially, to a different metabolic cellular program. We recently showed that it is possible to reprogramme *in vitro* CD8^+^ T cells form people on ART so that they acquire features associated with natural HIV control, by targeting specific metabolic pathways. These results suggest the potential interest of metabolic interventions as immunotherapies to achieve HIV remission.

**Added value of this study**

We show that rosuvastatin promotes a memory-like profile and enhanced the survival, proliferation, and cytokine polyfunctionality of HIV-specific CD8^+^ T cells from individuals on ART. These results indicate that it is possible to reprogramme *in vivo* HIV-specific CD8^+^ T cells by targeting the cholesterol pathway with rosuvastatin. Our results also suggest that statin treatment could be used, in combination with other immunotherapies, to enhance the antiviral capacity of CD8^+^ T cells.

**Implications of all the available evidence**

In addition to reprogramme CD8^+^ T cells *in vitro* for adoptive therapy approaches, direct statin administration could be used to enhance the functionality of HIV-specific cells, in strategies aiming at HIV remission. Moreover, the effects of metabolic reprogramming of CD8^+^ T cells with rosuvastatin could be extended to other viral infections. Our study is timely as the recent results of the REPRIEVE clinical trial have shown that daily statin treatment lowers the risk of cardiovascular disease in people with HIV receiving ART, and statins will likely be included in the new recommendations for managing HIV infection.

## Introduction

CD8^+^ T cells are critical for the natural or spontaneous control of human immunodeficiency virus (HIV) infection.^1^ Indeed, HIV natural controllers develop highly functional HIV-specific CD8^+^ T cells that contribute to effective control of viraemia in the absence of antiretroviral treatment (ART).^2^ The strong antiviral potential of HIV-specific CD8^+^ T cells from controllers is linked to a memory-like profile,^3^ comprising high survival, proliferative potential, and polyfunctionality.^4^, ^5^, ^6^, ^7^ Moreover, the early establishment of such memory-like CD8^+^ T cells appears to be a key stage towards natural control of infection.^8^ In contrast, skewed effector/exhausted-like differentiation of HIV-specific CD8^+^ T-cells is found in most people with HIV (PWH) who require ART.^6^, ^7^, ^8^

We recently showed that at least some of the differences in the capacities of HIV-specific CD8^+^ T cells from HIV controllers and other PWH are tied to their different metabolic programs,^7^ and that metabolic pathways can be targeted *in vitro* to confer capacities associated with natural control into cells obtained from HIV non-controllers.^9^ Cell metabolism is a central regulator of CD8^+^ T cell development and function.^10^ For instance, exhausted CD8^+^ T cells exhibit a skewed metabolism compared to functional effector cells. Some of these metabolic changes are induced and regulated by inhibitory receptors such as programmed death-1 (PD-1), and its blockade can at least partially restore the metabolic attributes and functionality of exhausted CD8^+^ T cells.^10^ Thus, restoration of cellular metabolism is a critical aspect to be considered in immunotherapies aiming at reversing T cell dysfunction in HIV infection.

The cholesterol pathway has been implicated in the induction of inhibitory receptors and acquisition of an exhausted T cell profile, dampening antitumour immunity.^11^ Cholesterol synthesis, downstream of the l-mevalonate pathway, can be decreased by 3-Hydroxy 3-methylglutaryl-coenzyme A (HMG-CoA) reductase inhibitors, also known as statins, which are commonly used to treat hypercholesterolaemia. Statins possess anti-inflammatory properties and have demonstrated safety and beneficial effects in clinical trials for autoimmune diseases.^12^ Statins have been evaluated in the context of HIV infection, showing a global reduction in the levels of activated CD8^+^ T cells from PWH on ART.^13^^,^^14^ Recently, the REPRIEVE study demonstrated that taking a daily statin lowered the risk of major adverse cardiovascular events in PWH with low to moderate risk of atherosclerotic cardiovascular disease,^15^ through lipidic and non-lipidic effects.^16^ Besides, results from preclinical models have demonstrated that statins also enhance CD8^+^ T cell functionality, promote antitumour activities, and potentiate the response to immune checkpoint blockade.^17^, ^18^, ^19^ Thus, metabolic reprogramming with statins is a promising strategy to enhance CD8^+^ T cell functionality.

We postulate that statins may exert an additional beneficial effect on HIV-specific CD8^+^ T cells from PWH on ART, by reducing the levels of inhibitory receptors, restoring metabolic alterations, and potentiating their functionality. The IMEA 043-CESAR trial was a pilot study that assessed the impact of high-dose rosuvastatin for 12 weeks on immune activation and inflammation in PWH on ART.^14^ Here, we performed an in-depth evaluation of the impact of rosuvastatin on the phenotype, metabolism, memory potential, and functionality of total and HIV-specific CD8^+^ T cells from the IMEA 043-CESAR participants. We found that rosuvastatin promoted a memory-like profile and enhanced the survival, proliferation, and cytokine polyfunctionality of total and HIV-specific CD8^+^ T cells. Our results shed light on the potential of *in vivo* metabolic reprogramming with statins to enhance the antiviral potential of HIV-specific CD8^+^ T cell.

## Methods

### Participants and study design

**Fig. 1:**
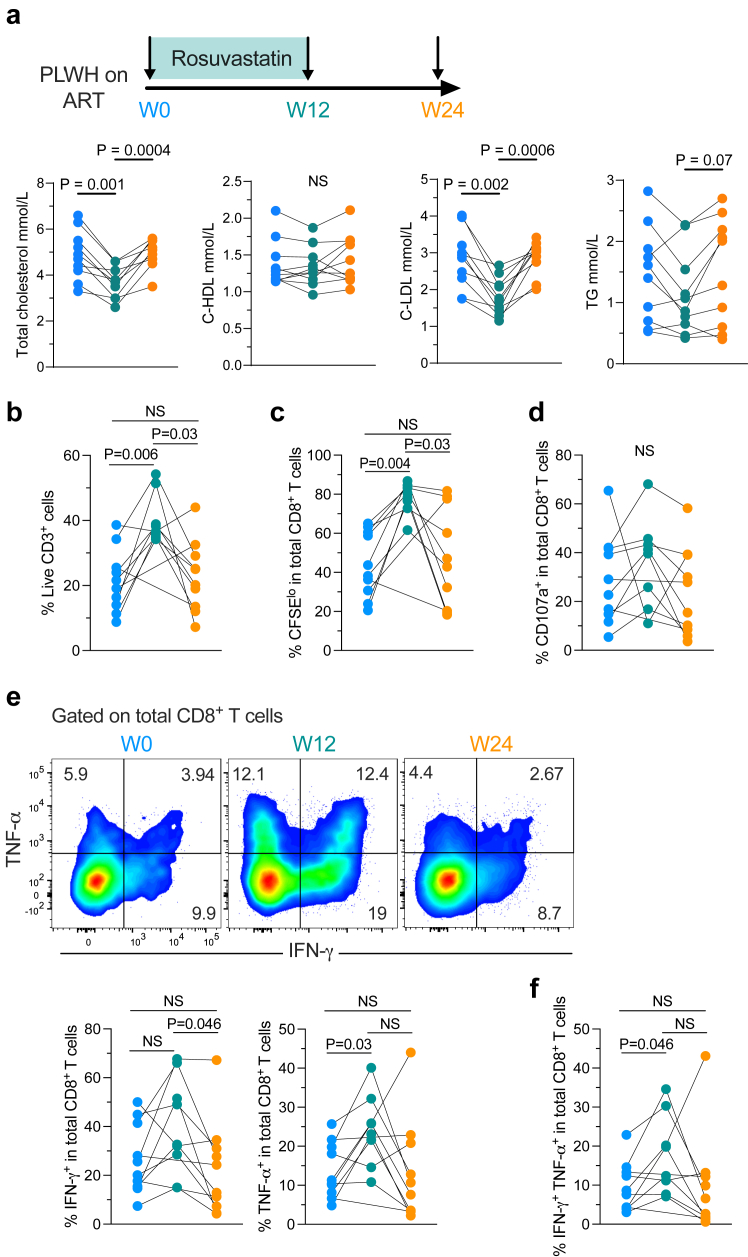
**Rosuvastatin promotes polyfunctional CD8^+^ T cells**. **a.** Study design and changes in total, HDL and LDL cholesterol, and triglycerides (TG) in the participants included in this study. **b–d.** Frequency of live CD3^+^ cells (**b**), as well as CFSE^lo^ (**c**), and CD107a^+^ (**d**) CD8^+^ T cells upon 6 days stimulation with anti-CD3/CD28 antibodies. **e.** Representative expression of IFN-γ and TNF-α in polyclonally-stimulated CD8^+^ T cells from the same donor at the three time points evaluated, and summary of the frequency of IFN-γ^+^ and TNF-α^+^ CD8^+^ T cells. **f.** Frequency of IFN-γ^+^ TNF-α^+^ CD8^+^ T cells. Samples from 10 participants were analysed (b–f, n = 8 at S12). Data obtained from 3 independent experiments are shown. NS: Not statistically significant (p > 0.05). Mixed effects model for repeated measures and Šidák's multiple-comparison test was used.

We studied samples from PWH on ART who were originally included in the IMEA 043-CESAR trial (NCT01874743), a bicentric open-label Phase II pilot prospective study.^14^ The objective of the study was to evaluate the impact of a short course of high-dose rosuvastatin on markers of immune activation and inflammation in PWH on ART who did not have, otherwise, specific clinical indications for statin treatment. Three subgroups of participants (not activated/not inflamed; CD8 activated; inflamed) were identified after hierarchical classification based on proximal component analyses of the markers of systemic inflammation and T cell activation (%CD38+HLA-DR+CD8+, %CD38+CD8+, %HLA-DR+CD8+, %Ki-67+CD8+, %HLA-DR+CD4+, and %Ki-67+CD4+ for T-cell activation; and hsCRP, IL-6, sCD14, and D-dimers) measured at the baseline of the trial for each participant. Eligible participants (who maintained ART throughout the study) received rosuvastatin (20 mg/day) for 12 weeks. Blood samples analysed in the present study were taken at week 0 (W0), week 12 (W12), and week 24 (W24) upon study inclusion (Fig. 1a). We included in this sub-study all the participants for whom at least two sequential samples were available with sufficient cells to perform the analyses (n = 10). Two W12 samples (participants 014 and 022) were not analysed due to poor viability or low number of cells upon thawing. Clinical data for participants included are summarised in Table S1. The group of individuals studied here was representative of the complete group of participants to the CESAR trial concerning demographic and clinical parameters such as sex (70% vs. 79% of men in this sub-study and the CESAR trial respectively, p = 0.68), age (48 [40–58] y vs.47 [41–54] y) or time on ART (5 [3–13] y vs. 7 [3–14] y). Sex was self-reported by study participants. All analyses were done on cryopreserved PBMCs isolated from blood samples.

### Ethics

The IMEA 043-CESAR study (NCT01874743) was approved by the ethic committee (comité de protection des personnes) of Ile de France XI. All participants gave their informed consent to participate in the study, and for their samples and data to be used for research purposes, according to the Helsinki declaration.

### Polyclonal and antigen-specific stimulation of CD8^+^ T cells

Thawed PBMCs were left rested for 6 h and subsequently split into two fractions. The first fraction was stimulated with plate-bound anti-CD3 and CD28 antibodies (both at 1 μg/mL; clones OKT3 (RRID: AB_468855) and CD28.2 (RRID: AB_468927), respectively; both from Thermo Fisher), and cultured for 48 h at 37 °C, 5% CO_2_, for subsequent analysis of metabolite uptake. The second fraction was employed in an experimental design aimed to reveal the CD8^+^ T cell survival and expansion capacity, a characteristic of memory-like cells. As such, cells were stained with carboxyfluorescein succinimidyl ester (CFSE; at 1 μM, Thermo Fisher Scientific), and next stimulated for 6 days with overlapping peptide pools encompassing HIV-1 consensus subtype B Gag protein (at 2 μg/mL; obtained through the National Institute of Health (NIH) AIDS Reagent Program, Division of AIDS, National Institute of Allergy and Infectious Diseases, NIH, catalogue number 12425), in the presence or absence of a combination of an anti-PD-1 (clone J116, RRID: AB_2573133, Thermo Fisher) and anti-LAG3 (clone 17B4, RRID: AB_11162489, Novus Biological) antibodies (both at 10 μg/mL). As a positive control, cells were stimulated with soluble anti-CD3 and CD28 antibodies (both at 1 μg/mL), while unstimulated cells were used as a negative control. Twelve h before completing the total incubation time, an additional dose of the peptides was added, as well as brefeldin A (10 μg/mL), monensin (1 μg/mL), and an anti-CD107a BV786 antibody, for subsequent analysis of intracellular cytokines. Polyclonally-stimulated cells were restimulated with PMA and ionomycin (50 and 500 ng/mL, respectively) also for 12 h, while unstimulated cells also received a 12 h stimulation with Gag peptides. In all the cases, the cells were cultured at 2 × 10^6^ cells/mL. No differences were observed in the level of cell viability after thawing measured by trypan blue staining (p = 0.3).

### Flow cytometry analysis

After culture, cells were stained with the LIVE/DEAD Fixable Aqua Dead Cell Stain kit (Thermo Fisher Scientific), with anti-CD3 APC eFluor 780, anti-CD8 BUV496, and anti-CD4 BUV737 antibodies. For phenotype analyses, cells were additionally stained with anti-CD45RA PE Cy7, anti-CCR7 PE Dazzle 594, anti-CD27 PE, anti-CD28 BV711, anti-PD-1 BUV661, anti-LAG3 BUV395, and anti-CD127 BV650 (gating strategy is shown in Fig. S7). Additional surface staining panels included anti-KLRG1 Alexa Fluor 700, anti-CD122 BV650, and anti-CXCR5 BV421. Next, cells were fixed and permeabilised with the BD Transcription Factor Buffer Set kit (BD Biosciences), for the detection of intranuclear proteins, or with the Phosflow fix/perm buffers (BD Biosciences), for the detection of phospho-proteins. Intracellular staining panels for the detection of transcription factors included anti-TCF-1 PE, anti-T-bet BV711, anti-Eomes PE eFluor 610, anti-TOX APC eFluor 660, and anti-BCL6 PE Cy7, as well as anti-IFNγ BV605 and anti-TNFα PerCP Cy5.5. Intracellular staining panels for detection of phospho-proteins included anti-granzyme B Alexa Fluor 700, anti-phospho-S6 S235/236 Pacific blue, anti-phospho-AKT S473 Alexa Fluor 647, and anti-IFNγ and anti-TNFα antibodies. The list of flow cytometry antibodies used in this study is provided in Table S2. Cell acquisition was performed using an LSR Fortessa X-20 flow cytometer (BD Biosciences), and data were analysed with FlowJo software (version 10, BD Biosciences). At least 10,000 live CD8^+^ T cells were acquired for each sample.

### Measurement of metabolite uptake

The analyses were performed on a subset of six participants for whom additional stored samples were available. Upon culture, PBMC were split into two parts and put into contact with 2-NBDG (2-(N-(7-Nitrobenz-2-oxa-1,3-diazol-4-yl)Amino)-2-Deoxyglucose) (150 μM, 30 min) for glucose uptake measurement, or BODIPY 500/510 C_1_, C_12_ (4,4-Difluoro-5-Methyl-4-Bora-3a,4a-Diaza-s-Indacene-3-Dodecanoic acid) (5 μM, 5 min) for fatty acid uptake measurement (both from Thermo Fisher Scientific). After these incubations, cells were stained with the LIVE/DEAD Fixable Aqua Dead Cell Stain kit, as well as with phenotype antibodies (as described above), and with an anti-GLUT1 PE or an anti-CD36 PE antibody. Finally, cells were immediately acquired on a FACS ARIA III flow cytometer (BD Biosciences).

### Statistical analyses

GraphPad Prism software version 9.0 was used for statistical analysis. Data are presented as medians and ranges. The mixed effects model for repeated measures with the Geisser-Greenhouse correction was used for the comparison of three or more paired groups. The Šidák's method was applied to correct for multiple comparisons. All p values less than 0.05 were considered as significant. Considering the small sample size, multivariate analyses were judged not adapted for this exploratory sub-study.

### Role of the funding source

The study sponsors did not have any role in study design, data collection, analysis, or interpretation, in the writing of the report, and in the decision to submit the paper for publication.

## Results

### Rosuvastatin promotes polyfunctional CD8^+^ T cells with enhanced survival

We included ten participants from the IMEA 043-CESAR trial with available stored sequential samples. All the individuals had CD4^+^ T cell counts of less than 500 cells/μL, indicative of incomplete immune reconstitution. In addition, they were classified according to their T cell activation and systemic inflammation status, as not activated/not inflamed, or activated/inflamed, as previously reported.^14^ The participants received rosuvastatin for 12 weeks, in addition to continuous ART. T cell analyses were performed at W0, W12, and W24 of the study corresponding to rosuvastatin treatment initiation, last dose of rosuvastatin, and 12 weeks off rosuvastatin, respectively (Fig. 1a). Baseline characteristics are described in Table S1. In agreement with the results reported for the main study, rosuvastatin treatment was accompanied by a decrease of the levels of total (p = 0.001, p = 0.0004) and LDL cholesterol (p = 0.002, p = 0.0006) at W12 (vs. W0 and W4 respectively) in all participants studied here (Fig. 1a). All the markers returned to baseline levels at W24. These results confirm the activity of statins at the different stages of the study protocol for the participants included here.

We focused our analyses on exploring the memory potential and stemness of CD8^+^ T cells, as these characteristics have been associated with natural control of HIV infection.^5^^,^^7^ First, we analysed the survival capacity and functionality of total CD8^+^ T cells, in terms of proliferation (%CFSE^lo^), degranulation (%CD107a^+^), and cytokine production (interferon [IFN]-γ and tumour necrosis factor [TNF]-α) upon 6-days anti-CD3/CD28 stimulation. Cells obtained from samples at W12 were characterised by enhanced viability, relative to samples at W0 (p = 0.006) (Fig. 1b). This effect returned to baseline levels at W24 (p = 0.03 and p = 0.96, W24 vs. W12 and W0, respectively) (Fig. 1b). A similar pattern was observed for the frequency of proliferating CD8^+^ T cells (p = 0.004 and p = 0.03, W12 vs. W0 and W24, respectively; p = 0.91 W24 vs. W0) (Fig. 1c). Increased expression of IFN-γ and TNF-α were observed at W12 vs. W24 (p = 0.05) or W0 (p = 0.03) respectively (Fig. 1e) but differences were not significant for CD107 (Fig. 1d). Of note, we observed a robust increase in the frequency of bifunctional IFN-γ^+^ TNF-α^+^ CD8^+^ T cells on the samples at W12 (p = 0.05 vs. W0) (Fig. 1e and f). All these effects waned, once rosuvastatin treatment was discontinued (p = 0.85, p = 1, p = 1, W24 vs. W0 for cells producing IFN-γ, TNF-α or both) (Fig. 1e and f). We explored if the effects of rosuvastatin also extended to CD4^+^ T cells and the results were less pronounced in comparison with CD8^+^ T cells (Fig. S1).

The effect of rosuvastatin on CD8^+^ T cells was not related to changes in the relative frequency of memory or effector subsets (Fig. S2A). However, all memory subsets benefited to some extent from the effects of rosuvastatin, with increases in the frequencies of CFSE^lo^, CD107a^+^, IFN-γ^+^, TNF-α^+^, and/or IFN-γ^+^ TNF-α^+^ cells being observed in W12 samples (Fig. S2B–F). Cells with a central memory phenotype experienced a general increase for all the functional markers evaluated. We again observed that the effect of rosuvastatin on memory CD8^+^ T cells was transient, as the frequencies of responding CD8^+^ T cells generally returned to baseline levels at W24 (Fig. S2B–F).

Some participants in the IMEA 043-CESAR study exhibited at baseline an activated/inflamed status (Table S1).^14^ We thus explored if the effects of rosuvastatin on CD8^+^ T cell functionality depended on the activation/inflammation status of the participants. Although comparisons were limited by the low number of samples, CD8^+^ T cells from individuals with an activated/inflamed status exhibited at baseline lower survival, proliferation capacity, and cytokine production after polyclonal stimulation (Fig. S3). Rosuvastatin enhanced the functional properties, while a decrease in the expression of PD-1 in CD8^+^ T cells was observed for all paired samples analysed independently of the participant status (W12 vs. W0, Fig. S3). Nevertheless, CD8^+^ T cells from activated/inflamed individuals appeared to maintain a lower functional capacity and higher PD-1 expression after 12 weeks of rosuvastatin treatment but the interpretation of these results is limited due to the low number of participants in each group (Fig. S3).

### CD8^+^ T cell-intrinsic metabolic changes upon rosuvastatin treatment

**Fig. 2:**
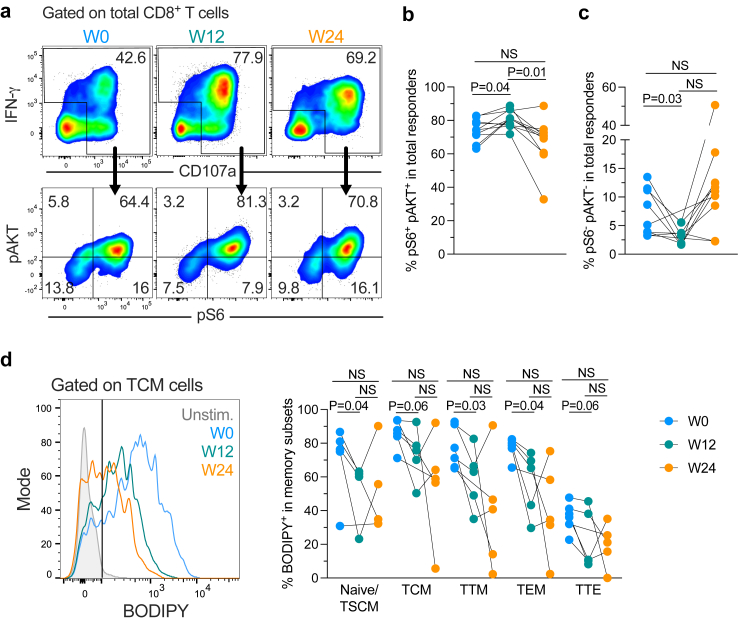
**Metabolic changes in CD8^+^ T cells upon rosuvastatin treatment**. **a–c.** PBMCs were stimulated for 6 days with anti-CD3/CD28 antibodies. Representative expression of pS6 and pAKT in total CD8^+^ responder cells (IFN-γ^+^ and/or CD107a^+^) from the same donor at the three time points evaluated (**a**), and summary of the frequency of pS6^+^ pAKT^+^ (**b**) and pS6^−^ pAKT^−^ (**c**) cells among total responder cells. Samples from 10 participants were analysed (n = 8 at S12). **d.** CD8^+^ T cells were stimulated for 48 h with anti-CD3/CD28 antibodies, and memory subpopulations were analysed. Representative expression of BODIPY on central memory CD8^+^ T cells and summary of the frequency of BODIPY^+^ among memory CD8^+^ T cells (n = 6 participants). Data obtained from two independent experiments are shown. NS: Not statistically significant (p > 0.05). Mixed effects model for repeated measures and Šidák's multiple-comparison test was used.

Cholesterol affects several metabolic pathways and induces cellular stress in CD8^+^ T cells, impairing their functionality.^11^ Thus, we hypothesised that rosuvastatin can modulate several metabolic pathways in CD8^+^ T cells. As such, we evaluated the impact of rosuvastatin on the mTORC pathways, as well as on glucose and fatty acid uptake. These three pathways regulate CD8^+^ T cell function and fate.^10^ We first evaluated in CD8^+^ total responder cells (i.e., producing IFN-γ^+^ and/or CD107a^+^ upon anti-CD3/CD28 stimulation for 6 days) the expression of phospho-S6 S235/236 (pS6) and phospho-AKT S473 (pAKT), markers of the activation of mTORC1 and mTORC2 pathways, respectively (Fig. 2a). We observed higher frequencies of pS6^+^ pAKT^+^ cells at W12 vs. W0 (p = 0.04) or vs. W24 (p = 0.01) (Fig. 2b). This was associated with a decrease in pS6^−^ pAKT^−^ cells at W12 vs. W0 (p = 0.03) (Fig. 2c). Thus, rosuvastatin treatment favours the synergistic activation of mTORC1 and mTORC2 pathways on polyclonally-activated CD8^+^ T cells.

We next evaluated if rosuvastatin treatment had any incidence on the uptake of fatty acids and glucose by CD8^+^ T cells. We did not observe changes in the expression of the fatty acid receptor CD36 (Fig. S4A). In contrast, we observed lower levels of BODIPY (indicator of fatty acid uptake) by CD8^+^ T cells on the W12 samples when compared to W0, and this was consistent across all memory CD8^+^ T cell subsets (p = 0.04, Naïve/TSCM; p = 0.06, TCM; p = 0.03, TTM; p = 0.04, TEM; p = 0.06, TTE) (Fig. 2d). In addition, we evaluated 2-NBDG (indicator of glucose uptake) along with the expression of the glucose transporter 1 (GLUT1) without observing significant differences (Fig. S4B). These results suggest that enhanced functionality provoked by rosuvastatin treatment is associated with less fatty acid greedy CD8^+^ T cells with synchronous mTORC activation.

### Rosuvastatin-induced polyfunctional CD8^+^ T cells exhibit a memory-like profile

**Fig. 3:**
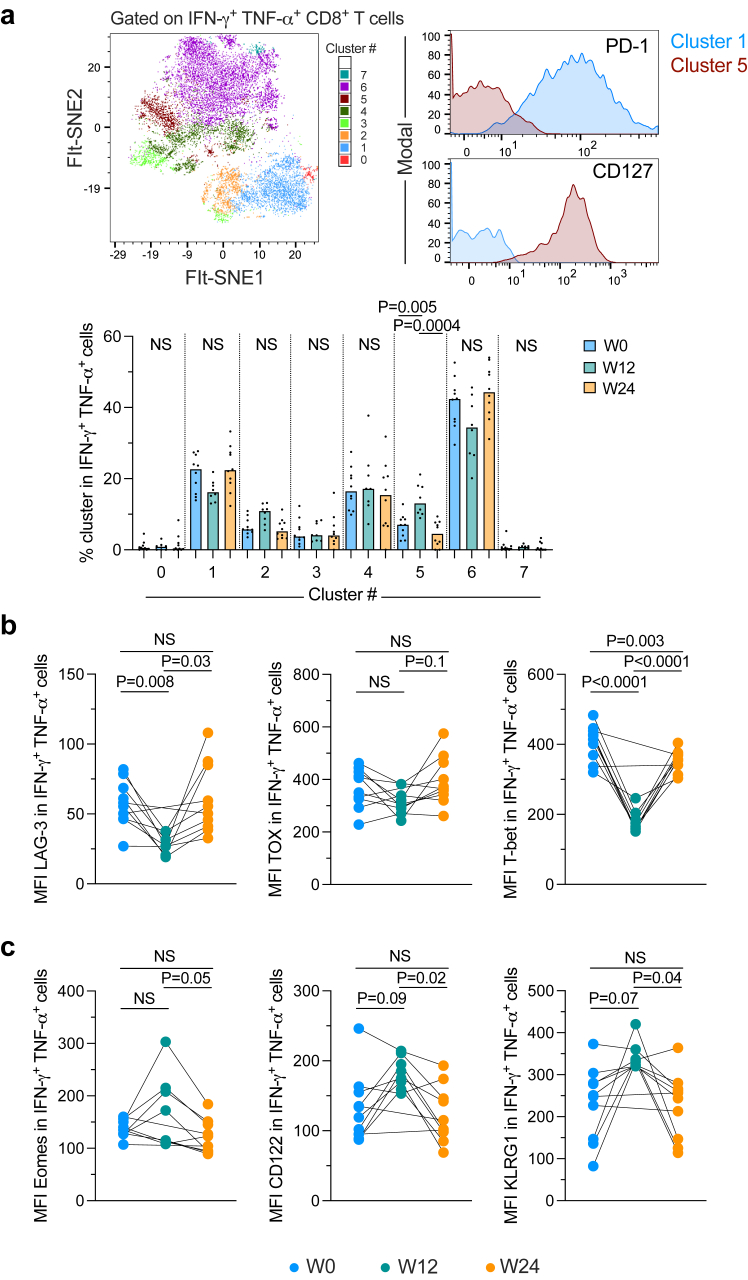
**A less effector/exhausted-like profile of polyfunctional CD8^+^ T cells upon rosuvastatin treatment**. **a.** FlowSOM clusters derived from IFN-γ^+^ TNF-α^+^ polyclonal CD8^+^ T cells were projected on a FIt-SNE plot. The expression of PD-1 and CD127 in clusters 1 and 5 are shown in the right panels. The frequency of each cluster among IFN-γ^+^ TNF-α^+^ cells in the samples analysed is shown in the bottom panel. **b.** Expression of LAG-3, TOX, and T-bet in IFN-γ^+^ TNF-α^+^ CD8^+^ T cells at the three time points evaluated. **c.** Expression of Eomes, CD122, and KLRG1 in IFN-γ^+^ TNF-α^+^ CD8^+^ T cells. Samples from 10 participants were analysed (b and c, n = 8 at S12). Data obtained from 3 independent experiments are shown. NS: Not statistically significant (p > 0.05). Mixed effects model for repeated measures and Šidák's multiple-comparison test was used.

We then explored if the increase in polyfunctional IFN-γ^+^ TNF-α^+^ CD8^+^ T cells observed after rosuvastatin treatment was accompanied by changes in markers of T cell activation, differentiation, and exhaustion. We performed unsupervised clustering using FlowSOM of the IFN-γ^+^ TNF-α^+^ CD8^+^ T cells detected in all the samples analysed (Fig. 3a). Eight clusters were identified, of which only the frequency of cluster 5 significantly changed after rosuvastatin treatment (p = 0.005 and p = 0.0004, W12 vs. W0 and W24 respectively) (Fig. 3a). Cluster 5 was characterised by the concomitant low expression of PD-1 and high expression of CD127 (Fig. 3a and Fig. S4C), consistent with a memory-like, less-exhausted phenotype.^20^ Accordingly, although changes for the T cell exhaustion-associated transcription factor TOX were not significant, IFN-γ^+^ TNF-α^+^ CD8^+^ T cells identified at W12 of rosuvastatin treatment exhibited lower expression of the inhibitory receptor LAG-3 (p = 0.008 and p = 0.03; W12 vs. W0 and W24), as well as a strong decrease in the effector-like transcription factor T-bet (p < 0.0001 W12 vs. both W0 and W24) (Fig. 3b). In contrast, these cells were characterised by increased expression of the transcription factor Eomesodermin (Eomes) (p = 0.05), usually associated with the establishment of T cell memory,^21^ and the IL-15 receptor β-chain CD122 (p = 0.02) at W12 when compared to W24 (Fig. 3c). We did not observe changes for other memory-associated phenotypic markers and transcription factors such as CD28, CXCR5, TCF-1, or BCL-6. Intriguingly, we also observed an increase in KLRG1 (p = 0.04, W12 vs. W24), a marker that is typically associated with T cell differentiation and the acquisition of the effector functions.^22^ Of note, the KLRG1^+^ Eomes^+^ T-bet^lo^ profile that we found enriched among IFN-γ^+^ TNF-α^+^ CD8^+^ T cells at W12 of rosuvastatin treatment has previously been described in CD8^+^ T cells with enhanced cytotoxicity and responsiveness to IL-15.^23^ Collectively, our results indicate that 12 weeks of rosuvastatin treatment favoured the accumulation of CD8^+^ T cells with a memory-like, less-exhausted phenotype, with multipotent antiviral potential and enhanced capacity to respond to homoeostatic cues.

### Increase of polyfunctional memory-like HIV-specific CD8^+^ T cells after rosuvastatin treatment

**Fig. 4:**
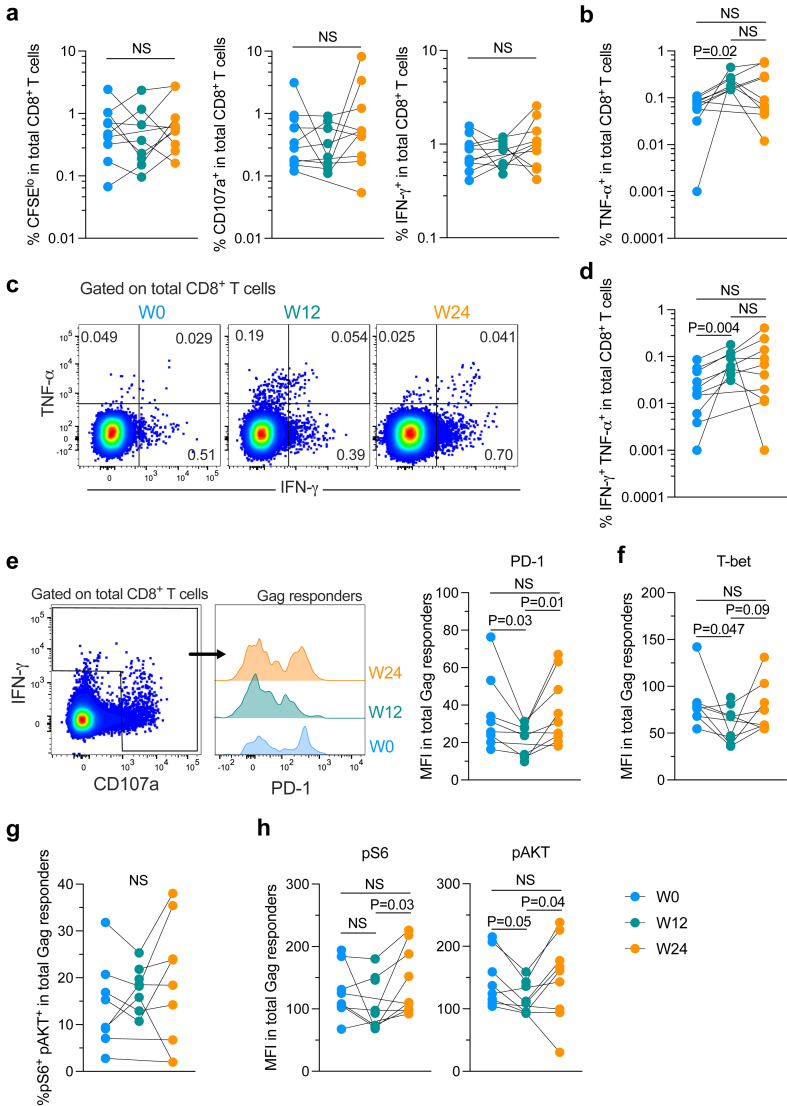
**Rosuvastatin promotes polyfunctional HIV-specific CD8^+^ T cells**. PBMCs were stimulated for 6 days with a pool of Gag peptides for the evaluation of HIV-specific CD8^+^ T cells. Frequency of CFSE^lo^, CD107a^+^, IFN-γ^+^ (**a**), and TNF-α^+^ (**b**) HIV-specific CD8^+^ T cells. **c.** Representative expression of IFN-γ and TNF-α in Gag-stimulated CD8^+^ T cells from the same donor at the three time points evaluated. **d.** Summary of the frequency of IFN-γ^+^ TNF-α^+^ HIV-specific CD8^+^ T cells. Data obtained from 3 independent experiments are shown. **e.** Left: Representative expression of PD-1 in CD8^+^ Gag responder cells (IFN-γ^+^ and/or CD107a^+^); Right: Summary of the expression of PD-1 in Gag responder CD8^+^ T cells. **f.** Summary of the expression of T-bet in Gag responder CD8^+^ T cells. **g.** Frequencies of pS6^+^ pAKT^+^ in Gag responder CD8^+^ T cells. **h.** Expression of pS6 and pAKT on a per-cell basis in Gag responder CD8^+^ T cells. Samples from 10 participants were analysed (n = 8 at S12). Data obtained from 3 independent experiments are shown. NS: Not statistically significant (p > 0.05). Mixed effects model for repeated measures and Šidák's multiple-comparison test was used.

We next sought to evaluate if rosuvastatin treatment impacted the functionality of HIV-specific CD8^+^ T cells. To evaluate the memory potential of HIV-specific CD8^+^ T cells, we sequentially stimulated circulating CD8^+^ T cells from samples at each timepoint with Gag peptides *in vitro* and evaluated the expansion capacity along with cytokine production of responding cells in a 6-day-interval experiment (Fig. S5A), as previously described.^9^^,^^24^^,^^25^ We did not observe changes in the frequency of CFSE^lo^ (p = 0.47), CD107a^+^ (p = 0.2), or IFN-γ^+^ (p = 0.27) CD8^+^ T cells responding to 6-days stimulation with HIV-1 Gag peptides at the different timepoints of the study (Fig. 4a). However, an increase in the frequency of TNF-α^+^ (p = 0.02) (Fig. 4b) and IFN-γ^+^ TNF-α^+^ CD8^+^ T cells (p = 0.004) (Fig. 4c and d) was found at W12 relative to W0, in line with the results obtained on polyclonally stimulated cells, and this effect seemed to be sustained at W24 (p = 1 and p = 0.83, W12 vs. W24 for TNF-α^+^ and IFN-γ^+^ TNF-α^+^ CD8^+^ T cells respectively). The increase in TNF-α^+^ CD8^+^ T cells induced by rosuvastatin was independent of the activated/inflamed status of the individual (Fig. S5B). Thus, rosuvastatin treatment potentiates polyfunctional abilities in HIV-specific CD8^+^ T cells, while preserving their proliferation and degranulation capacity.

We next evaluated the phenotypic characteristics of HIV-specific CD8^+^ T cells after rosuvastatin treatment that could be linked to their increased polyfunctionality. Total Gag-specific CD8^+^ T cells from samples at W12 contained a higher proportion of less-effector/exhausted cells, given by a lower expression of PD-1 (p = 0.03, p = 0.01 relative to W0 and W24) and T-bet (p = 0.05, relative to W0) (Fig. 4e). In addition, Gag-responder CD8^+^ T cells maintained the frequencies of pS6^+^ pAKT^+^ cells in all timepoints (p = 0.41) (Fig. 4g), but they exhibited lower levels of pS6 (p = 0.03, W12 relative to W24) and pAKT (p = 0.05 and p = 0.04, W12 relative to W0 and W24 respectively) on a per-cell basis (Fig. 4h). We also focused our analysis on TNF-α-producing CD8^+^ T cells in response to Gag-peptides, which were more frequent after 12 weeks of rosuvastatin treatment (the analysis of IFN-γ^+^ TNF-α^+^ cells was not performed given the low number of events). Consistent with the results on polyclonal cells, TNF-α^+^ Gag-specific CD8^+^ T cells from samples at W12 contained a higher proportion of less-effector/exhausted cells, given by a lower expression of LAG-3 (p = 0.004 and p = 0.001) and T-bet (p = 0.001 and p = 0.001), relative to W0 and W24 (Fig. S5C). These data further support the notion that HIV-specific CD8^+^ T cells acquire a memory-like, less-effector/exhausted phenotype upon rosuvastatin treatment.

Statins may enhance the response to immune checkpoint blockade and therapeutic efficacy in cancer models.^17^, ^18^, ^19^ We therefore explored if HIV-specific CD8^+^ T cells from samples at W12 of rosuvastatin treatment were more able to respond to immune checkpoint blockade. We stimulated CD8^+^ T cells for 6 days with Gag peptides in the absence or simultaneous presence of anti-PD-1 and anti-LAG-3 antibodies and evaluated the frequency of TNF-α^+^ cells. Of note, we did not observe an effect of PD-1/LAG-3 blockade on HIV-specific cells in any of the samples at W0 and, in some cases, we observed further impairment of the response in the presence of the antibodies (Fig. S6A and B). In contrast, we observed that PD-1/LAG-3 blockade increased the frequency of TNF-α^+^ Gag-specific CD8^+^ T cells in 3 out of 8 W12 samples (Fig. S6A and B). These 3 responders were the participants 009 (not activated/not inflamed group), 005 (activated/inflamed group), and 001 (not assigned to any of these groups; Table S1). An analysis of TNF-α^+^ Gag-specific CD8^+^ T cells from the 3 responder individuals at W12 revealed a trend for a lower expression of LAG-3 on a per-cell basis as well as a lower frequency of pS6^+^ cells, in comparison with cells from non-responder individuals, while no difference was observed at W0 (Fig. S6C). Although these analyses are strongly limited by the low number of individuals studied, these results might imply that the response to immune checkpoint blockade could be enhanced by rosuvastatin in some PWH.

## Discussion

We report that a short-term, high-dose rosuvastatin treatment in PWH on ART and incomplete immune reconstitution confers higher polyfunctionality and survival of total and HIV-specific CD8^+^ T cells, associated with the modulation of some metabolic features, the decrease of inhibitory receptors and the promotion of a memory-like profile. This effect was independent of the baseline CD8^+^ T cell differentiation state, as well as the activated/inflamed status of the individual.

Statins have been used in PWH receiving ART to decrease the risk of cardiovascular disease and residual T cell activation and inflammation.^13^, ^14^, ^15^ In addition, statins exert direct effects on T cells, particularly associated with their activation and differentiation status. Cholesterol is a key component of cell membranes and is especially found in lipid rafts, which contain multiple T cell signalling molecules.^26^ In line, previous studies have demonstrated that statins alter cholesterol distribution in the cell membrane,^27^ as well as the membrane localisation and activation of GTPases, thus modulating TCR signalling.^28^ Additional mechanisms of statins to modulate T cell activation include the blockade of integrin signalling,^29^ and modulation of antigen presentation.^30^ Collectively, these direct and indirect effects of statins may affect the molecular program of CD8^+^ T cells and alleviate persistent antigen and cytokine stimulation, contributing to reducing their dysfunction during chronic infections.^31^

Memory-like CD8^+^ T cells have strong antiviral potential and therapeutic benefit in settings of chronic antigen stimulation, related to their enhanced survival capacity, preservation of polyfunctionality, and multi-lineage differentiation.^20^ Interestingly, we observed that rosuvastatin globally promoted the survival and polyfunctionality of CD8^+^ T cells in individuals with HIV, independently of their basal CD8^+^ T-cell activation. These improved functional properties were associated with the promotion of a less effector and exhausted-like profile, as well as metabolic plasticity of CD8^+^ T cells, which were able to sustain their functions with a lower metabolite consumption. The modulation of metabolism and the acquisition of a memory-like profile are both critical to induce and sustain the CD8^+^ T cells with enhanced antiviral and antitumour potential.^9^^,^^32^ In line, previous studies in tumour models have demonstrated that reducing cholesterol *in vivo* with statins enhances the persistence of antitumour CD8^+^ T cells.^33^ The enhanced antitumour activity of CD8^+^ T cells induced by statins may also be associated with reduced endoplasmic reticulum stress response and lower expression of immune checkpoints.^11^ These effects are particularly beneficial in environments enriched in cholesterol, such as in peripheral tissues (where the major HIV replication occurs) and tumours.^34^

Interestingly, while we found that most effects of rosuvastatin were similarly observed on polyclonal and HIV-specific CD8^+^ T cells, there were a few differences. In particular, whereas anti-CD3/CD28-responder cells observed after 12 weeks of rosuvastatin treatment showed an increased capacity to synchronously mobilise mTORC1 and mTORC2 pathways, HIV-specific cells were characterised at the same time by lower per-cell levels of these metabolic pathways without diminishing their capacity to proliferate or produce cytokines. These divergent features among CD8^+^ T cells are likely related to the strength and duration of TCR signalling they received *in vivo*. Indeed, dysfunctional cells from PWH on ART receive strong and/or persistent antigen stimulation, linked to higher activation of the mTORC1 pathway.^7^ Continuous mTORC1 activation has been associated with an effector-like profile and poor memory potential.^35^ In contrast, non-HIV-specific CD8^+^ T cells are expected to have a less dysfunctional signature. Likely, the maintenance of a more quiescent profile during rosuvastatin treatment may facilitate CD8^+^ T cell activation, engagement of mTORC-related anabolic pathways, and sustain their effector functions. Importantly, the higher polyclonal CD8^+^ T cell activation during rosuvastatin treatment was not associated with the acquisition of exhaustion traits. In contrast, activated cells maintained a memory-like profile, with higher CD127 expression and less LAG-3 and T-bet.

Here we also show that not all CD8^+^ T cell functions are equally modulated by rosuvastatin. Indeed, TNF-α production was particularly enhanced in total and HIV-specific cells, an effect that seemed to be sustained at W24, while this was not observed for IFN-γ production or the degranulation capacity alone. Indeed, the memory-like, less-exhausted profile promoted by rosuvastatin treatment was particularly enriched among TNF-α^+^ cells. These results are in keeping with our previous studies,^7^^,^^9^ where we showed that TNF-α production is associated with a superior functionality and memory potential of HIV-specific cells. Thus, our data further support the notion that TNF-α production is a relevant marker of memory-like CD8^+^ T cells and can be a useful indicator of the effectiveness of novel immunotherapies targeting this cell subset.

Several characteristics promoted by rosuvastatin in HIV-specific CD8^+^ T cells have been previously associated with efficient responses in natural HIV controllers,^4^^,^^5^^,^^7^ and could be a complement in CD8^+^ T cell-based strategies for an HIV cure. Indeed, we observed that rosuvastatin appeared to facilitate the *in vitro* response to immune checkpoint blockade in some participants, an effect that may be related to the memory-like profile exhibited by these cells, as well as the reduced expression of PD-1 and LAG-3.^20^ Supporting this assumption, prior work has identified that a CD8^+^ T cell subset with an intermediate expression of PD-1 and low LAG-3 confers a response to immune checkpoint blockade in the setting of chronic stimulation.^36^ Moreover, the higher survival of CD8^+^ T cells induced by rosuvastatin could also improve the persistence of adoptively transferred cells.^37^ Finally, in addition to the effects on CD8^+^ T cells, reducing cholesterol with rosuvastatin could also limit HIV replication in host cells,^38^ which would be beneficial in shock and kill strategies to prevent novel infections.

A limitation of our study is the small number of individuals analysed, and the non-placebo-controlled design, which increases the uncertainty of the analyses realised. In addition, due to sample availability, we were not able to evaluate the profile of cells specific for other viral antigens and the effect of rosuvastatin in tissue CD8^+^ T cells. In contrast a major strength of our study is the *in vivo* evaluation of the effect of cholesterol synthesis inhibition on the functionality of CD8^+^ T cells. In addition, we evaluated sequential samples, before, during, and at the distance of statin treatment. This experimental design allowed us to define the transient effects of rosuvastatin on CD8^+^ T cells, strongly supporting the direct influence of the treatment. The precise molecular mechanism by which rosuvastatin improves HIV-specific CD8^+^ T cell functionality remains to be explored. The CESAR trial was conducted almost ten years ago, and it is unclear whether similar effects would be observed in actual conditions of ART, where treatment is often based on integrase inhibitors and started earlier. A rosuvastatin dose of 20 mg daily was chosen in the CESAR trial based on the results of the JUPITER trial,^39^ which demonstrated a reduction of major cardiovascular events in a large population of HIV negative individuals with elevated high-sensitivity C-reactive protein levels but without hyperlipidaemia. Future studies should evaluate whether the same effect could be observed with doses of rosuvastatin commonly used in clinical settings (10 mg/day), as well as the impact of other statins on HIV-specific CD8^+^ T cell functionality.

In summary, here we show that HIV-specific CD8^+^ T cell metabolic reprogramming with rosuvastatin enhances their functionality and, at least partially, potentiates their memory potential. Other metabolic interventions, such as administration of metformin or semaglutide have been shown to reduce inflammation and/or improve immune function in PWH without diabetes, underlining the effect of metabolism in the immune response in this context.^40^^,^^41^ Although it is unlikely that statin treatment by itself will be sufficient to promote HIV control in the context of ART interruption, CD8^+^ T cell *in vivo* metabolic reprogramming with rosuvastatin could be used to potentiate the therapeutic efficacy of this cell population in immunotherapies aiming at HIV cure or remission, as it has been recently described in the case of PD-1 inhibitors for the treatment of cancer.^42^^,^^43^ In addition, the benefits of CD8^+^ T cell metabolic reprogramming with rosuvastatin could be extended to the context of other chronic viral infections, where antigen-specific CD8^+^ T cells have a skewed and dysfunctional profile.

## Contributors

FP-C and AS-C conceived the study. FP-C, CP, and VM performed experiments and analysed the data. OL contributed to the conception and discussion of results. MFC, DC, and LW participated in participants recruitment and design of the clinical trial. FP-C and AS-C wrote the manuscript. All authors revised the manuscript. LW and AS-C provided financial support and supervised the work. FP-C and AS-C have accessed and verified the underlying data. All authors read and approved the final version of the manuscript.

## Data sharing statement

All data are available in the main text or the Supplementary Materials. Additional deidentified individual data will be made available upon reasonable request to the corresponding author (asier.saez-cirion@pasteur.fr), contingent on approval by the study's scientific committee and in alignment with ethical considerations.

## Declaration of interests

None to declare.
