# Supplementary material for: Impact of rosuvastatin on the memory potential and functionality of CD8+ T cells from people with HIV
Source: eBioMedicine. 2025 Mar 29;114:105672. doi: 10.1016/j.ebiom.2025.105672 (PMC11995788; doi:10.1016/j.ebiom.2025.105672)
Supplement: Figs and Tables [file mmc1.pdf]

Supplementary Materials

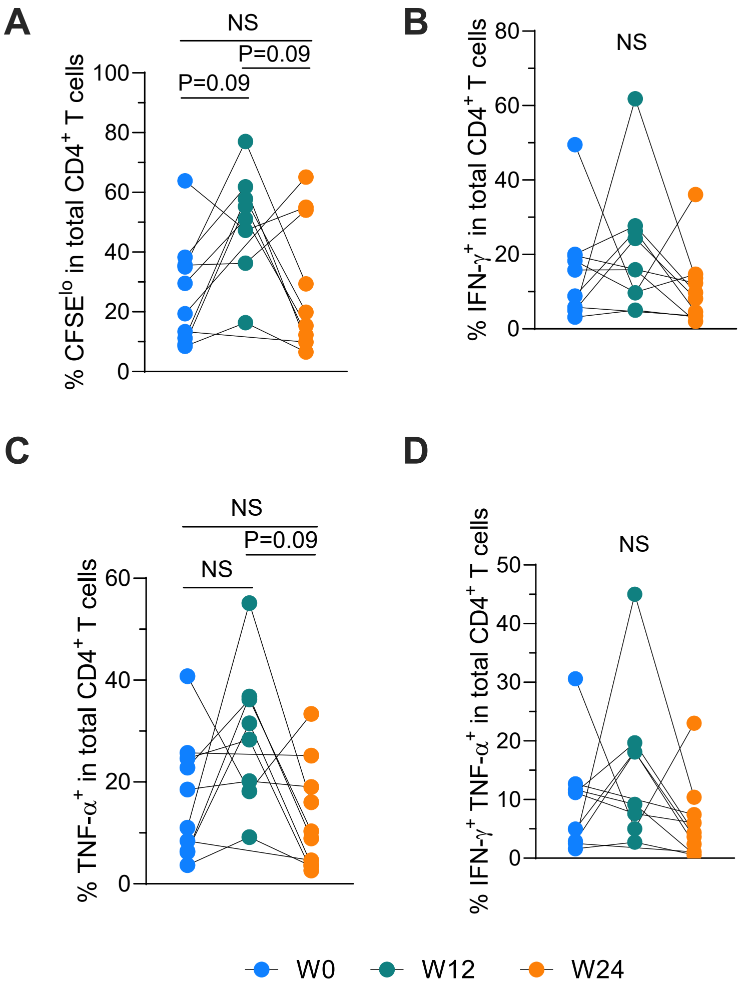

Fig. S1. Impact of statin treatment on CD4+ T cells. Frequency of $CFSE^{lo}$ (**A**), IFN-γ^+ (**B**), TNF-α^+ (**C**), and IFN-γ^+ TNF-α^+ (**D**) in polyclonally-stimulated $CD4^+$ T cells. Samples from 10 participants were analyzed (n=8 at S12). Data were obtained in 3 independent experiments. NS: Not statistically significant. Mixed effects model for repeated measures and Šidák's multiple-comparison test was used.

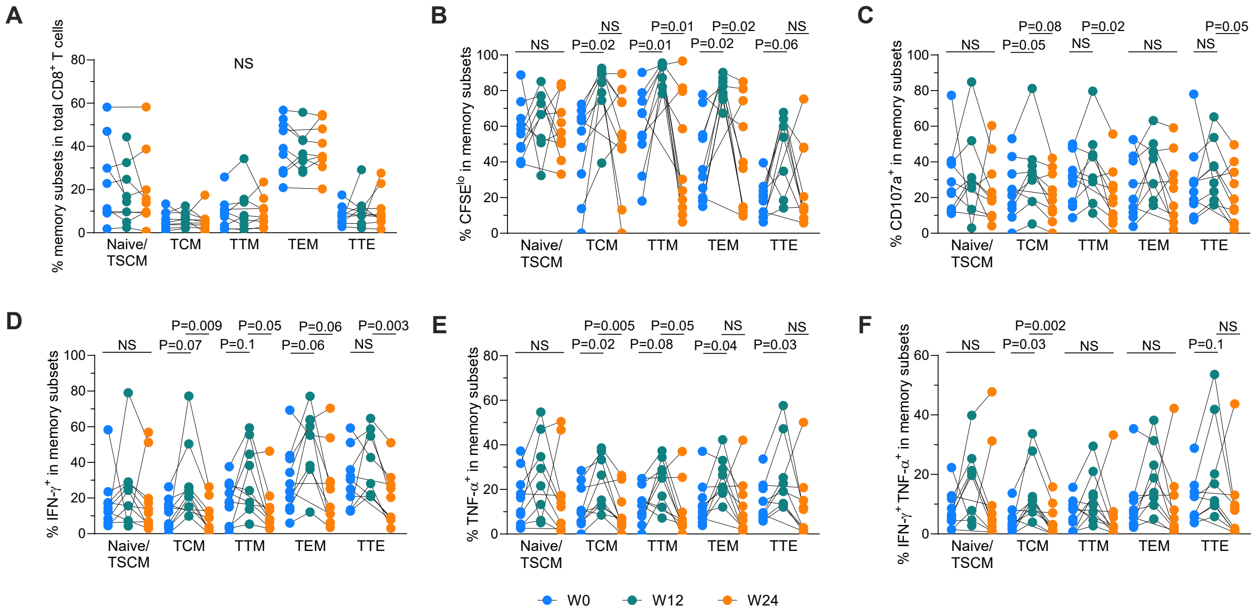

Fig. S2. Enhanced polyfunctionality of CD8+ T cell memory subsets upon treatment with rosuvastatin. A. Frequency of memory subsets in total unstimulated CD8^{+} T cells. **B-E.** CD8^{+} T cells were stimulated for 6 days with anti-CD3/CD28 antibodies, and memory subpopulations were analyzed. Frequency of CFSE^{lo} (**A**), CD107a^{+} (**B**), IFN-γ^{+} (**C**), TNF-α^{+} (**D**), and IFN-γ^{+} TNF-α^{+} (**E**) memory CD8^{+} T cells upon stimulation. Samples from 10 participants were analyzed (n=8 at S12). Data were obtained in 3 independent experiments. NS: Not statistically significant. Mixed effects model for repeated measures and Šidák's multiple-comparison test was used. Naïve/Stem cell memory (TSCM): CD45RA^{+} CCR7^{+} CD27^{+}; Central memory (TCM): CD45RA^{-} CCR7^{+} CD27^{+}; Transitional memory (TTM): CD45RA^{-} CCR7^{-} CD27^{+}; Effector memory (TEM): CD45RA^{-} CCR7^{-} CD27^{-}; Terminal effectors (TTE): CD45RA^{+} CCR7^{-} CD27^{-}.

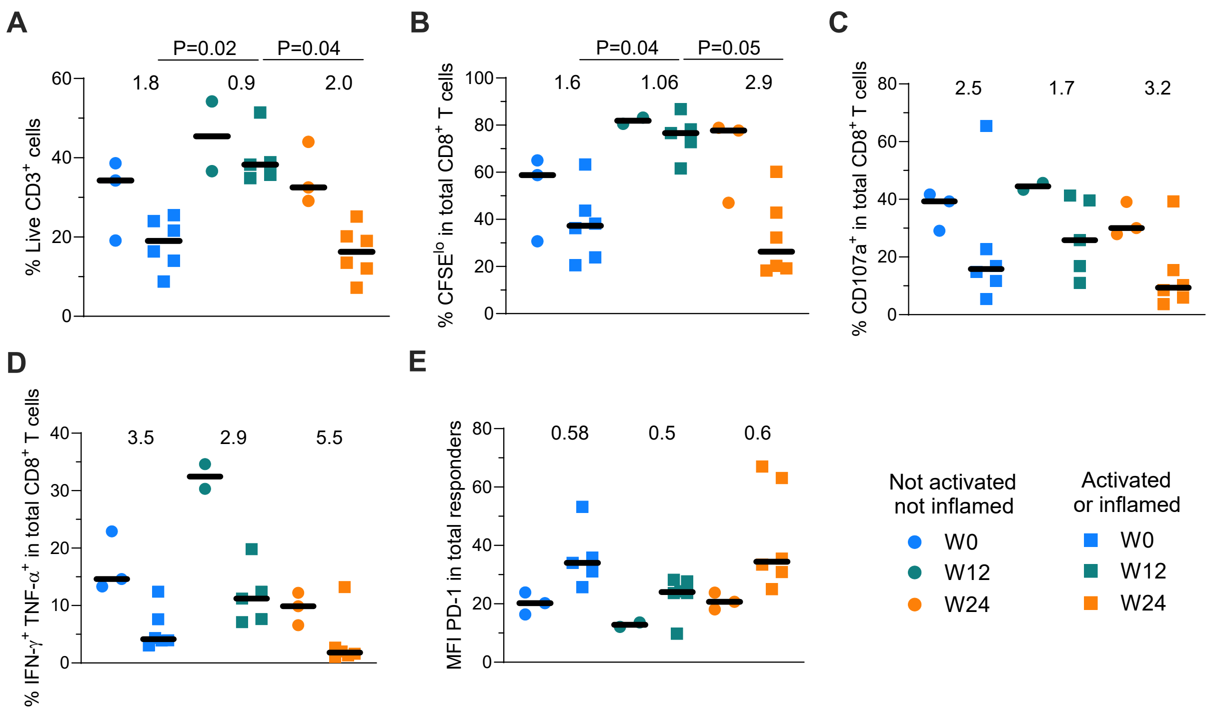

Fig. S3. Changes in CD8$^+$ T cell features in individuals classified according to the activated/inflamed clinical profile. The frequency of live CD3$^+$ cells (**A**), as well as CFSE^{lo} (**B**), CD107a$^+$ (**C**), IFN-γ$^+$ TNF-α$^+$ (**D**), and PD-1 expression (median fluorescence intensity, MFI) (**E**) in total CD8$^+$ T cells upon anti-CD3/CD28 stimulation was evaluated. Samples from 9 participants (3 classified as not activates/not inflamed and 6 activated/inflamed) were analyzed. Data obtained from 3 independent experiments are shown. The number above each time point indicates the mean difference between not activated/not inflamed versus activated/inflamed individuals. Mixed effects model for repeated measures and Šidák's multiple-comparison test was used.

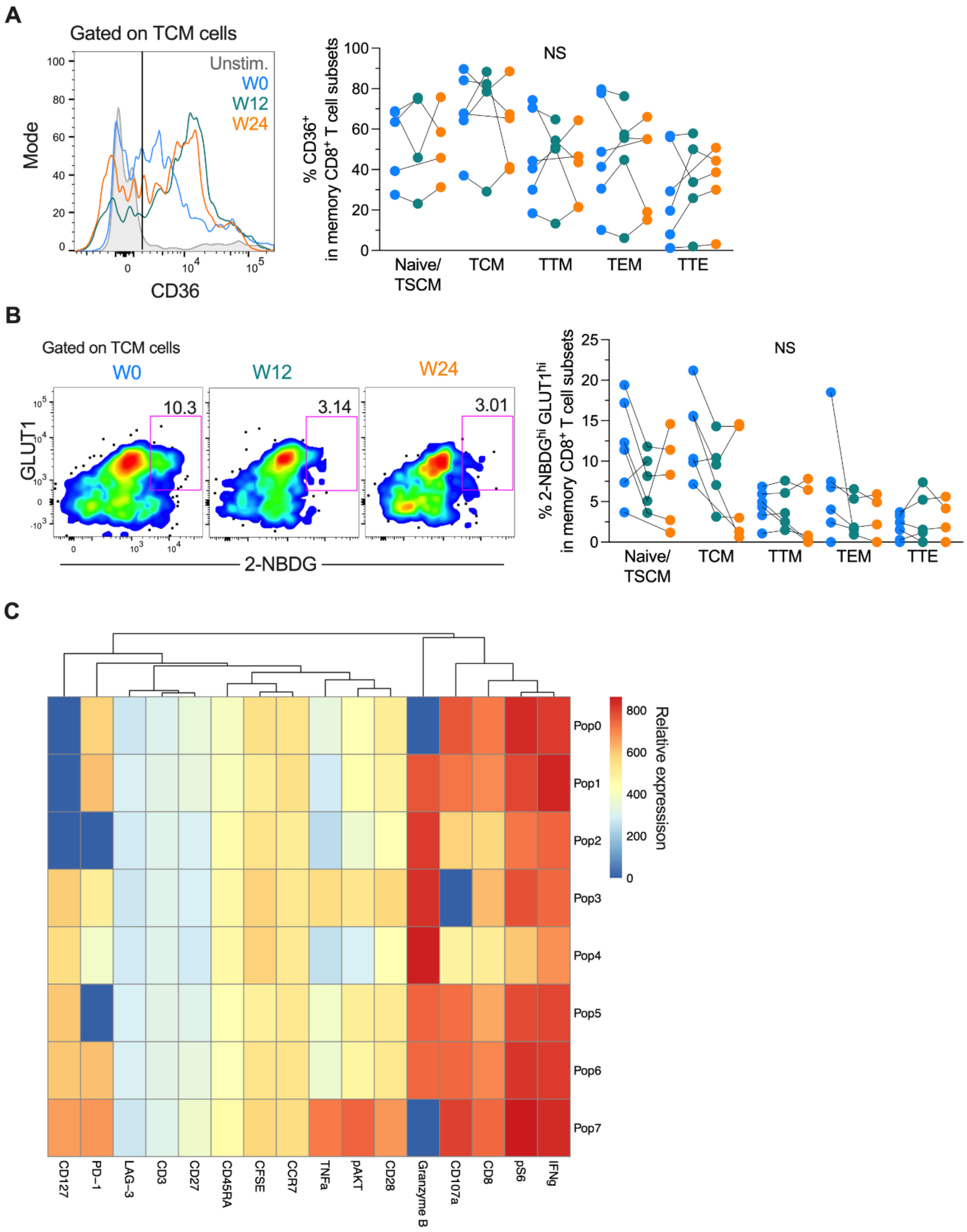

Fig. S4. Analyses on CD8+ T cell subsets. $CD8^+$ T cells were polyclonally stimulated for 48 hs, and memory subpopulations were analyzed. **A.** Left: Representative expression of CD36 in central memory cells. Right: Summary of the frequencies of $CD36^+$ cells. **B.** Left: Representative expression of GLUT1 and 2-NBDG in central memory cells. Right: Summary of the frequencies of GLUT-1^{hi} 2-$NBDG^{hi}$ cells. Samples from 6 participants were analyzed. Data were obtained from two independent experiments. NS: Not statistically significant. Mixed effects model for repeated measures and Šidák's multiple-comparison test was used. **C.** Heatmap generated from the FlowSOM analysis of IFN-γ^+ TNF-α^+ polyclonal $CD8^+$ T cells. The relative expression of each of the markers in the clusters identified is shown.

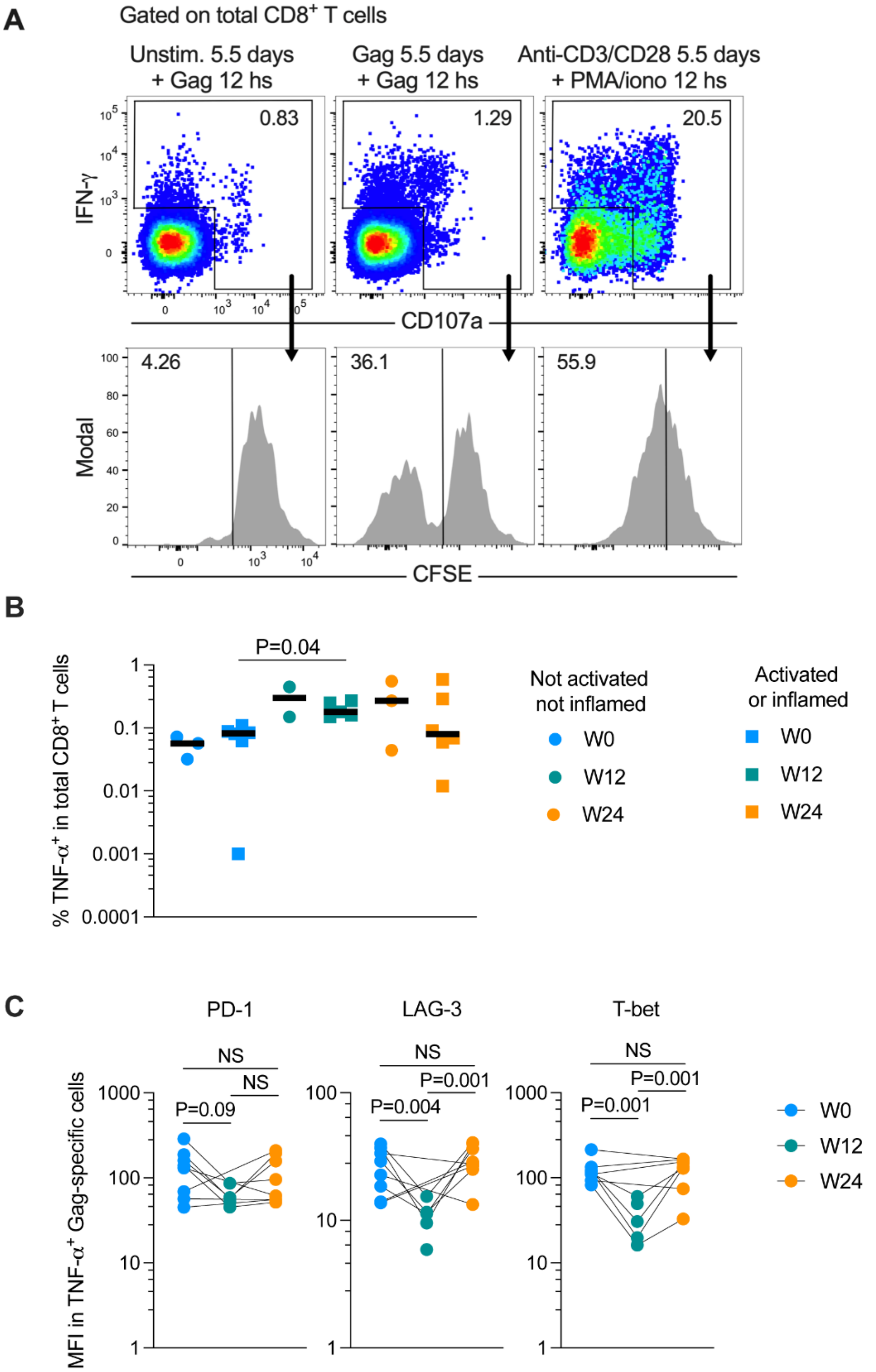

Fig. S5. Analyses of HIV-specific CD8+ T cell responses. **A.** Representative plots showing Gag-responder cells after 6-day stimulation. The magnitude of CFSElo cells for each condition is shown below. **B.** Frequency of TNF-α$^+$ HIV-specific CD8$^+$ T cells in individuals classified according to the activated/inflamed clinical profile. Samples from 9 participants (3 classified as not activates/not inflamed and 6 activated/inflamed) were analyzed. **C.** Summary of the expression of PD-1, LAG-3, and T-bet in TNF-α$^+$ Gag-specific CD8$^+$ T cells. Samples from 10 participants were analyzed (n=8 at S12). Data were obtained from 3 independent experiments. NS: Not statistically significant. Mixed effects model for repeated measures and Šidák's multiple-comparison test was used.

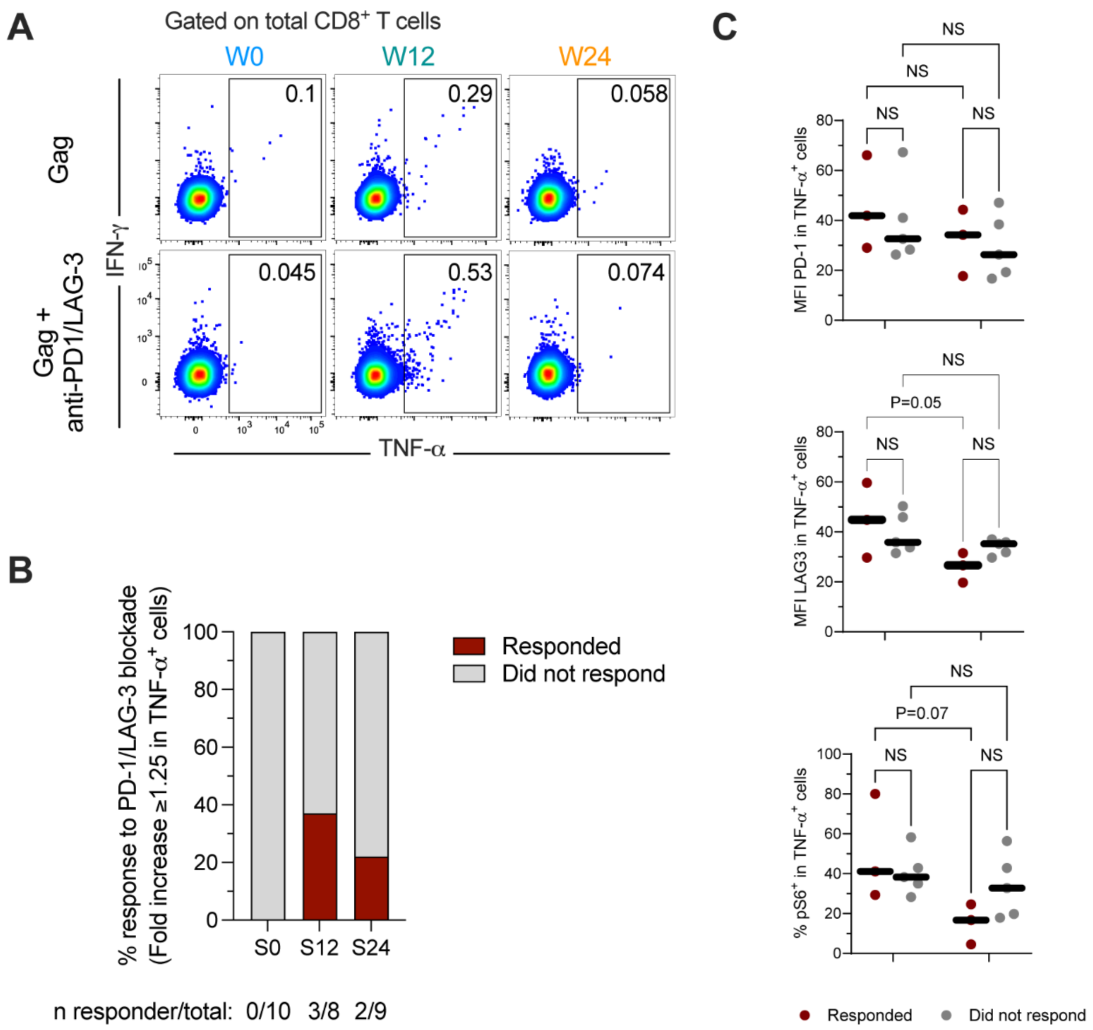

Fig. S6. Impact of statins on responsiveness of CD8+ T cells to PD-1/LAG-3 blockade in vitro. A. Representative expression of IFN-γ^+ and TNF-α^+ upon stimulation with Gag peptides alone or with anti-PD-1 and anti-LAG-3 antibodies, for 6 days. Data obtained at the three time points from the same donor are shown. **B.** Rate of response to PD-1/LAG-3 blockade at each time point. **C.** Expression of PD-1 and LAG-3, as well as the frequency of pS6$^+$ cells among TNF-α^+ cells upon 12 hs stimulation with Gag peptides, at W0 and W12. The individuals are classified as those who responded (n=3) or did not respond (n=5) to PD-1/LAG-3 blockade in vitro at W12. Data were obtained from 3 independent experiments. NS: Not statistically significant. A two-way mixed-effects model for repeated measures was used for the analysis.

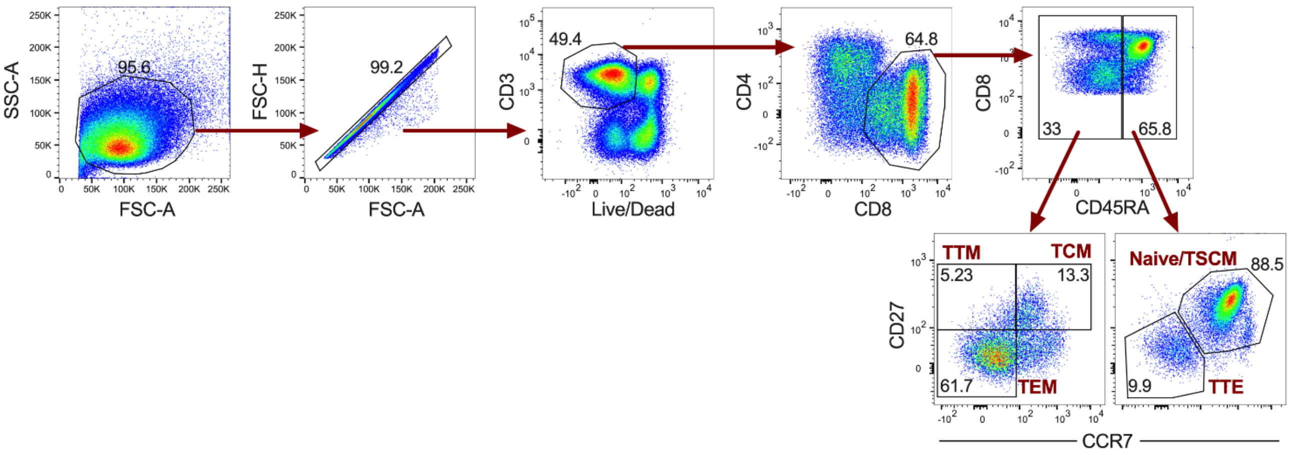

Fig. S7. Gating strategy to identify memory T cell subsets. naïve/Stem cell memory (TSCM): $CD45RA^{+}$ $CCR7^{+}$ $CD27^{+}$; Central memory (TCM): $CD45RA^{-}$ $CCR7^{+}$ $CD27^{+}$; Transitional memory (TTM): $CD45RA^{-}$ $CCR7^{-}$ $CD27^{+}$; Effector memory (TEM): $CD45RA^{-}$ $CCR7^{-}$ $CD27^{-}$; Terminal effectors (TTE): $CD45RA^{+}$ $CCR7^{-}$ $CD27^{-}$.

Table S1. Baseline clinical characteristics of the study cohort.

Group	Donor ID	Age (years)	Sex	ART duration (months)	Nadir $CD4^+$ T cells/µL	$CD4^+$ T cells/µL	$CD8^+$ T cells/µL	CD4/CD8 ratio	%$HLA\text{-}DR^+$ $CD38^+$ $CD8^+$ T cells	%$Ki\text{-}67^+$ $CD8^+$ T cells	CRP, mg/L	Soluble CD14, ng/mL
1	004	54.8	Male	29	36	222	567	0.39	0.78	1.87	1.00	1450
	009	63.1	Male	139	37	247	453	0.55	1.48	1.85	0.43	1970
	022	57.9	Male	27	33	260	908	0.29	0.64	1.51	0.72	1410
2	003	41.6	Male	49	131	173	437	0.4	3.19	2.40	7.44	1440
	005	59.1	Male	190	92	279	627	0.44	4.20	3.91	5.97	1550
	014	33.6	Female	38	92	267	653	0.41	1.16	2.43	0.69	2210
	015	52.6	Male	35	67	233	1714	0.14	1.13	1.78	NA	NA
	017	43.6	Female	72	104	319	855	0.37	0.91	1.03	3.65	1965
	021	42.3	Male	197	47	317	1014	0.31	2.59	3.35	1.18	1470
3	001	34.1	Female	76	96	221	294	0.75	1.22	5.12	NA	NA

1 Not activated/not inflamed; 2 Activated/inflamed; 3 Not assigned. CRP: C-reactive protein. NA: Not available data.

Table S2. Flow cytometry antibodies.

Target	Fluorophore	Company	Clone	Cat number	RRID	Dilution 1/
CD3	APC eFluor 780	Thermo Fisher	UCHT1	47-0038-42	AB_1272042	50
CD8	BUV496	BD	RPA-T8	612942	AB_2870223	200
CD4	BUV737	BD	OKT4	750977	AB_2875046	200
CCR7	PE Dazzle 594	Biolegend	G043H7	353236	AB_2563641	40
CD27	PE	BD	M-T271	560985	AB_10563213	40
CD45RA	PE Cy7	BD	5H9	561216	AB_10611721	80
CD28	BV711	BD	CD28.2	563131	AB_2738020	80
CD127	BV650	BD	HIL-7R-M21	563225	AB_2738081	80
LAG-3	BUV395	BD	T47-530	745640	AB_2743137	80
PD-1	BUV661	BD	EH12.1	750260	AB_2874457	80
CD122 (IL-15R beta)	BV650	BD	MIK-Beta3	743117	AB_2741294	40
KLRG1	Alexa Fluor 700	Biolegend	SA231A2	367730	AB_2890802	40
CXCR5	BV421	BD	RF8B2	562747	AB_2737766	80
TCF-1	PE	BD	S33-966	564217	AB_2687845	20
T-bet	BV711	BD	04-46	563320	AB_2738136	80
TOX	eFluor 660	Thermo Fisher	TXRX10	50-6502-82	AB_2574265	80
BCL6	PE Cy7	BD	K112-91	563582	AB_2738292	50
Eomesodermin	PE eFluor 610	Thermo Fisher	WD1928	61-4877-42	AB_2574616	80
TNF-α	PerCP Cy5.5	BD	MAb11	560679	AB_1727579	40
Granzyme B	Alexa Fluor 700	BD	GB11	560212	AB_11154033	80
IFN-γ	BV605	BD	B27	562974	AB_2737926	40
CD107a	BV786	BD	H4A3	563869	AB_2738458	200
Phospho-AKT Ser 473	Alexa Fluor 647	Cell signaling	D9E	4075S	AB_916029	50
Phospho-S6 S235/236	Pacific blue	Cell signaling	D57.2.2E	8520S	AB_2797646	50
GLUT1	PE	Abcam	EPR3915	ab209449	AB_3676092	200
CD36	PE	Biolegend	5-271	336206	AB_2072513	40
